# Vedolizumab use for severe chronic enterocolitis in a child with Hoyeraal–Hreidarsson syndrome: A case report

**DOI:** 10.1002/jpr3.70291

**Published:** 2026-10-29

**Authors:** Yasutaka Chiba, Manabu Takahashi, Taro Yoshida, Seiya Tagane, Takeshi Shiohata, Shoko Miura, Manami Akasaka

**Affiliations:** ^1^ Department of Pediatrics Iwate Medical University Yahaba Japan

**Keywords:** gut‐selective therapy, *RTEL1* mutation, telomere biology

## Abstract

We report a patient with Hoyeraal–Hreidarsson syndrome (HHS), a telomere biology disorder, who developed severe chronic enterocolitis with inflammatory bowel disease‐like features. Vedolizumab led to improvement in stool frequency, reduced fecal calprotectin, and clinical remission without infectious complications. Remission was maintained with vedolizumab monotherapy for 2 years after discontinuation of budesonide and parenteral nutrition. This report highlights vedolizumab as a potentially safe therapeutic option for enterocolitis in HHS, emphasizing the value of gut‐selective therapy when systemic immunosuppression is risky. Further studies are needed to establish the long‐term efficacy and safety of vedolizumab in patients with telomere biology disorders.

## 1 INTRODUCTION

Hoyeraal–Hreidarsson syndrome (HHS), a severe form of dyskeratosis congenita, is characterized by profound telomere dysfunction and is associated with multisystem complications, including bone marrow failure, immunodeficiency, and gastrointestinal involvement.^1^ It typically presents in early childhood with growth retardation and cerebellar hypoplasia. Enterocolitis associated with HHS is often refractory to standard therapies for inflammatory bowel disease (IBD), including 5‐aminosalicylic acid (5‐ASA), corticosteroids, and tumor necrosis factor (TNF) inhibitors, and is difficult to manage.^2^ The diagnosis and initial clinical course of the same patient with HHS due to *RTEL1* mutations were previously reported in Pediatric Blood and Cancer.^3^ Herein, we describe the subsequent development of severe enterocolitis and its successful management with vedolizumab.

## 2 CASE REPORT

The patient was a male born at 29 weeks of gestation with intrauterine growth restriction and oligohydramnios, weighing 902 g. Because of small intestinal obstruction, jejunal anastomosis and enterostomy were performed on day 1 of life, and the stoma was closed at 3 months of age. Congenital cerebellar hypoplasia was identified, and he has gradually exhibited developmental delay during infancy. At 2 years, he presented with pancytopenia. Telomere shortening and genetic analyses identified *RTEL1* mutations, and a diagnosis of HHS was established.

At 5 years, he was admitted with fever and persistent bloody diarrhea lasting 1 month. His height and weight were 103.3 cm (–1.1 SD) and 13.5 kg (–1.7 SD), respectively. A physical examination revealed oral aphthous ulcers, nail dystrophy, and mild abdominal tenderness. Laboratory data showed pancytopenia (white blood cell 1,500/μL, hemoglobin 8.8 g/dL, platelet 22,000/μL), hypoalbuminemia (2.9 g/dL), elevated C‐reactive protein (11.12 mg/dL), and fecal calprotectin was 1130 µg/g. Colonoscopy revealed multiple ulcerative lesions distributed throughout the entire colon (Figure 1). Upper gastrointestinal endoscopy revealed small ulcers and erosions at both the gastroesophageal junction and gastric antrum (Figure 2). Histopathology demonstrated crypt distortion, epithelial apoptosis, and inflammatory cell infiltration without crypt abscesses or granulomas, supporting chronic enterocolitis with IBD‐like histopathological features. However, epithelial apoptosis may also reflect mucosal injury associated with the underlying telomere biology disorder. Based on the clinical, endoscopic, and histopathological findings, the patient was diagnosed with severe enterocolitis. The patient did not fulfill the diagnostic criteria for Crohn's disease or ulcerative colitis. Because small‐bowel evaluation was incomplete, the condition was diagnosed as severe chronic enterocolitis with IBD‐like features. Bowel rest with parenteral nutrition was initiated on hospital day 12, and enteral nutrition was gradually reintroduced thereafter. Considering the risk of severe infection, vedolizumab was administered at a dose of 6 mg/kg on hospital days 19, 33, and 61, followed by maintenance every 8 weeks. Vedolizumab administration was approved by our institutional ethics committee and written parental consent was obtained. Before initiating vedolizumab therapy, infectious disease screening was performed, including stool cultures, Clostridioides difficile toxin, HIV, hepatitis B virus, hepatitis C virus, Epstein–Barr virus, cytomegalovirus, and T‐SPOT.TB assay for tuberculosis, all of which were negative. From day 20, stool frequency decreased, and fecal calprotectin dropped to 86 µg/g on day 26. On day 48, the abdominal pain worsened and hematochezia increased; consequently, oral budesonide (3 mg/day) was added, prioritizing the avoidance of systemic immunosuppression while considering local efficacy and bioavailability. Because hematologic abnormalities could confound Pediatric Crohn's Disease Activity Index (PCDAI), disease activity was monitored using the Pediatric Ulcerative Colitis Activity Index (PUCAI). The PUCAI score was 65 on admission. Following vedolizumab treatment, the score decreased to 5 by day 60, indicating clinical remission (Figure 3). Subsequently, bloody stools recurred, and budesonide enemas (2 mg/day) were administered. After resumption of oral intake, remission was maintained with combined central venous and enteral nutrition, and the patient was discharged on day 108. After discharge, budesonide was discontinued on day 120, and parenteral nutrition was discontinued on day 176. Since then, remission has been maintained with vedolizumab monotherapy administered every 8 weeks without recurrence of enterocolitis. At the last follow‐up (day 656), the fecal calprotectin level was 82 µg/g.

**Figure 1:**
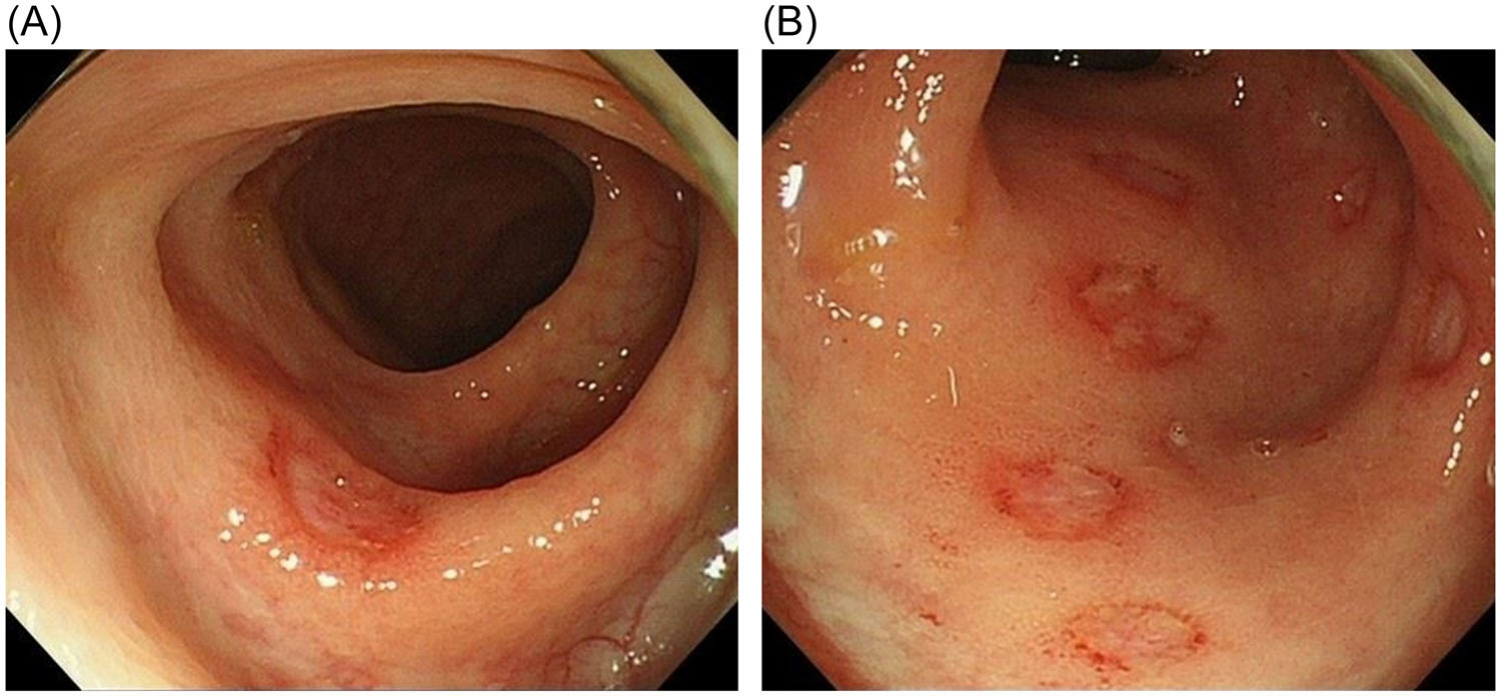
Colonoscopy findings. Colonoscopy revealed circular and partly longitudinal ulcerative lesions involving the entire colon.

**Figure 2:**
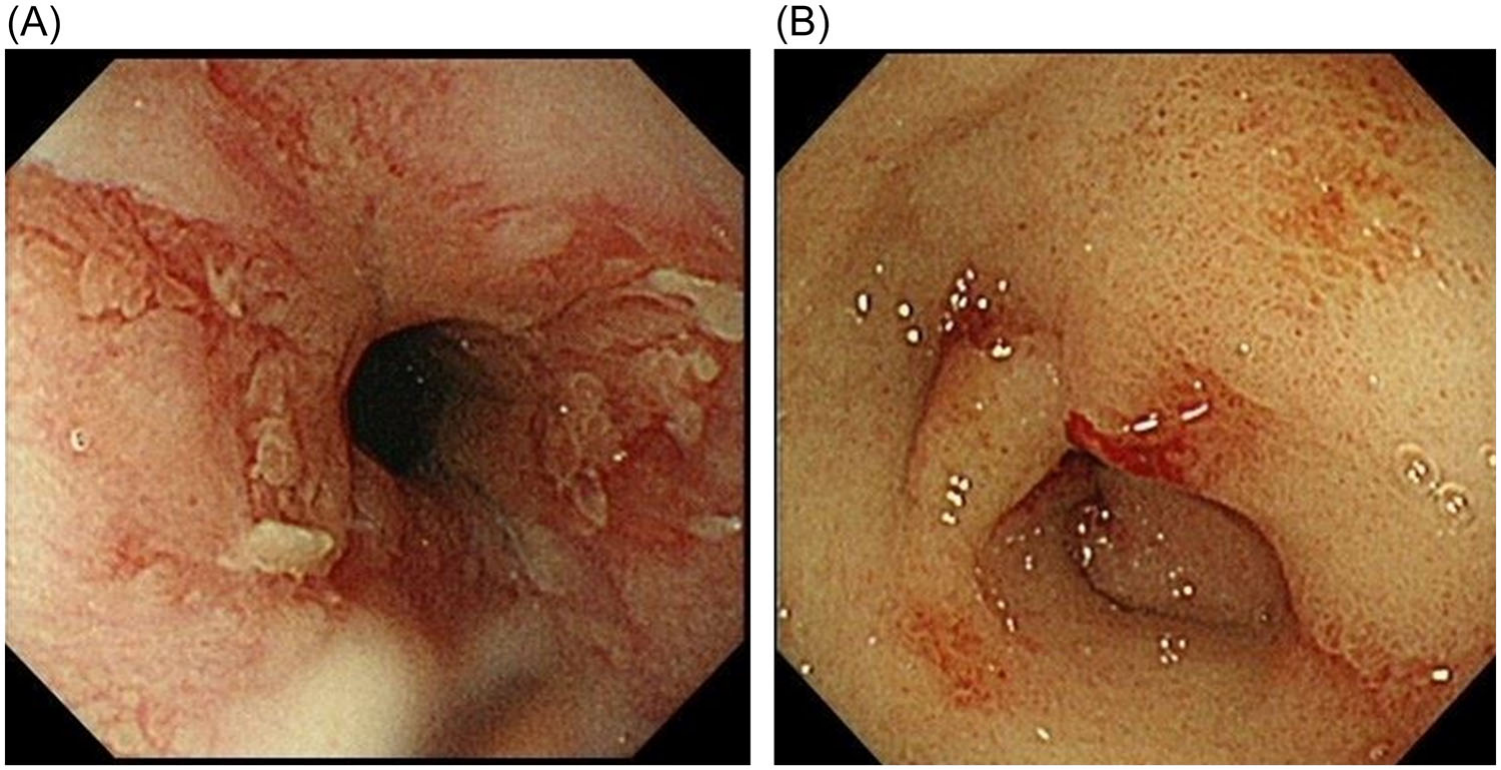
Upper gastrointestinal endoscopy findings. Upper gastrointestinal endoscopy revealed small ulcers and erosions at the gastroesophageal junction and gastric antrum.

**Figure 3:**
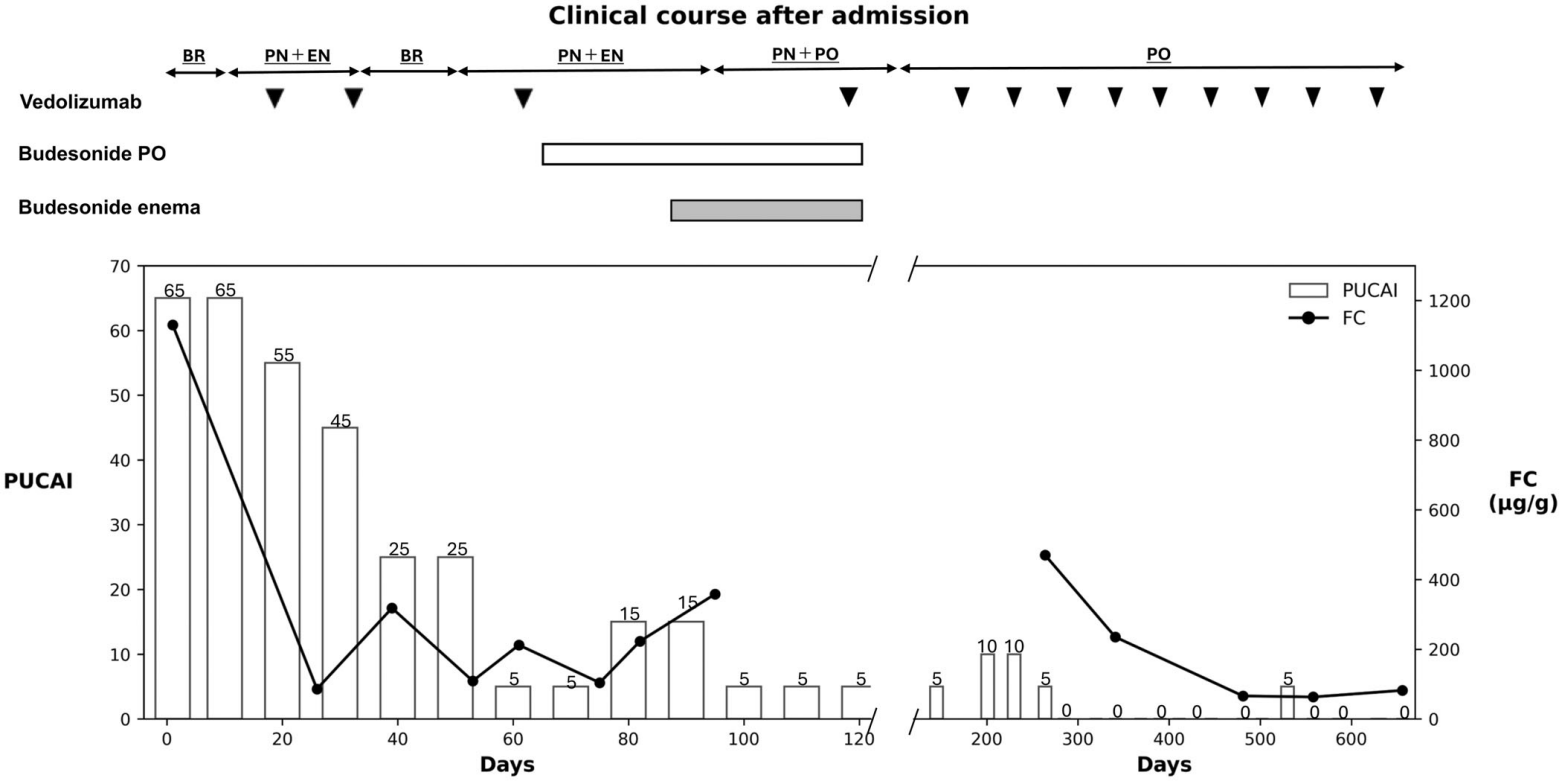
Clinical course after admission. Vedolizumab was administered on hospital days 19, 33, and 61, followed by maintenance therapy every 8 weeks. Oral budesonide was initiated on day 48, and budesonide enema was added after recurrence of bloody stools; both were discontinued on day 120. The figure shows the temporal relationship between treatment, nutritional management, the PUCAI, and FC levels. The clinical course is presented in two panels (≤120 days and >120 days) to illustrate the early treatment response and long‐term follow‐up. BR, bowel rest; EN, enteral nutrition; FC, fecal calprotectin; PN, parenteral nutrition; PO, oral intake; PUCAI, Pediatric Ulcerative Colitis Activity Index.

## 3 DISCUSSION

Allogeneic hematopoietic cell transplantation (HCT) is the only curative treatment for bone marrow failure and immunodeficiency. However, HCT does not improve organ dysfunction, such as pulmonary, hepatic, or gastrointestinal disease, and systemic complications can have a critical impact on prognosis.^1^ HHS‐related enterocolitis is considered to arise from exacerbated epithelial injury due to impaired DNA replication and telomere dysfunction.^1^, ^2^, ^4^, ^5^ Similar to monogenic IBD caused by single‐gene defects, it is commonly refractory to standard IBD therapies, such as 5‐ASA, corticosteroids, and anti‐TNF antibodies. Previous reports on *RTEL1*‐associated enterocolitis have documented its refractoriness to standard IBD treatments.^2^, ^6^ Patients with HHS exhibit combined immunodeficiency associated with telomere dysfunction, leading to increased susceptibility to infections. In such patients, the use of systemic immunosuppressive therapies may be associated with a higher risk of severe infections, making therapeutic decisions particularly challenging.

In this patient, TNF inhibitors were considered problematic because of concerns regarding infection risk and efficacy; therefore, vedolizumab, a gut‐specific agent, was selected, as it is expected to have minimal systemic immunosuppressive effects compared with conventional biologic therapies. Vedolizumab selectively inhibits α4β7 integrin‐mediated lymphocyte trafficking to the intestinal mucosa, thereby reducing intestinal inflammation while minimizing systemic immunosuppression.^7^, ^8^ Clinical trials have demonstrated favorable safety.^7^ In this case, clinical remission was achieved without any infectious complications. Vedolizumab may therefore represent a promising treatment for telomeropathy‐associated enterocolitis. Although initial clinical improvement may have been influenced by concomitant budesonide, sustained remission during 2 years of vedolizumab monotherapy supports its role in maintaining disease control.

## 4 CONCLUSION

We report a child with HHS who developed enterocolitis and was successfully treated with vedolizumab without major adverse events. This case underscores the potential of vedolizumab as a gut‐selective therapy for telomere disorders. Further studies are needed to establish its long‐term efficacy and safety.

## CONFLICT OF INTEREST STATEMENT

The authors declare no conflicts of interest.

## ETHICS STATEMENT

The authors acknowledge that written informed consent was obtained from the family for the use of the images included in this manuscript. Institutional review board approval was not required for this single case report.
